# Comparison of Outcomes Between McKeown and Sweet Esophagectomy in the Elderly Patients for Esophageal Squamous Cell Carcinoma: A Propensity Score-Matched Analysis

**DOI:** 10.1177/1073274820904700

**Published:** 2020-02-12

**Authors:** Dongni Chen, Yihuai Hu, Youfang Chen, Jia Hu, Zhesheng Wen

**Affiliations:** 1 Department of Thoracic Oncology, State Key Laboratory of Oncology in South China, Collaborative Innovation Center for Cancer Medicine, Sun Yat-sen University Cancer Center, Guangzhou, P. R. China

**Keywords:** esophageal squamous cell carcinoma, older patients, surgical approach, prognostic factor

## Abstract

The aim of this study was to compare the perioperative outcomes and long-term survival rates of the McKeown and Sweet procedures in patients with esophageal cancer younger than 70 years or older than 70 years. A total of 1432 consecutive patients with esophageal squamous cell carcinoma (ESCC) who received surgery at Sun Yat-sen University Cancer Center from January 2009 to October 2012 were analyzed. Propensity score matching was used to balance the clinical characteristics of the patients who underwent different surgical approaches, and 275 and 71 paired cases were matched among those younger and older than 70 years, respectively. The prognosis and postoperative outcomes were compared between the McKeown and the Sweet esophagectomy. For patients younger than 70 years, those who underwent the McKeown procedure had better overall survival (OS) than those in the Sweet group (log rank = 4.467; *P* = .035). However, no significant difference in disease-free survival and OS was observed between two approaches for the elderly patients (log rank = 1.562; *P* = .211 and log rank = 0.668; *P* = .414, respectively). Cox regression analysis revealed that McKeown approach was a positive prognostic factor compared to the Sweet approach for patients younger than 70 years in univariable analysis (HR = 0.790; 95% CI, 0.625-0.997; *P* = .047), whereas the surgical approach was not significantly related to the prognosis in the elderly patients. For patients older than 70 years, the occurrence of anastomotic fistula increased in those who underwent the McKeown procedure (23.9% vs 11.3%, *P* = .038, for the McKeown and Sweet esophagectomy, respectively). The McKeown approach increases the OS in younger patients with ESCC. However, for patients older than 70 years, the Sweet approach was proven to be an effective therapy, given the better perioperative outcomes and similar long-term survival compared with patients in the McKeown group.

## Introduction

Esophageal carcinoma (EC) ranked seventh in terms of incidence and sixth in mortality overall in 2018 worldwide.^1^ In China, esophageal cancer is the fifth most common cancer in males and is responsible for 9.9% of cancer-related deaths.^2^ Squamous cell carcinoma (SCC) and adenocarcinoma are the two most common histologic subtypes of EC, and SCC is the predominant histological type in China.^1^ Esophageal cancer tends to be diagnosed mainly in elderly men. As reported by Chen et al,^3^ the largest proportion of new cases with cancer and deaths in patients occurring in the age range from 60 to 74 years. Radical resection with lymphadenectomy remains the most important curative therapy. However, because of the high incidence of organ dysfunction and the aggressiveness of operative therapy, surgical indications for the elderly patients with EC remain unclear.^4^

Elderly EC patients are often recommended for palliative treatment, such as chemoradiotherapy (CRT), or endoluminal esophageal stent placement, considering that the operative mortality and comorbidities among the elderly patients were considerably higher than those of younger patients.^5,6^ Conversely, Bakhos et al^7^ reported that multimodality treatment did not confer a survival advantage compared to surgery alone in the elderly patients. In some previous studies, no significant differences were observed in the prognosis between the elderly and younger patients after esophagectomy,^4,8,9^ which indicated that the age should not be considered a contraindication to esophageal resection. However, the standard surgical approach for esophagectomy is unclear. In Western countries, the use of transhiatal versus transthoracic procedures is the major debate.^10^ Nevertheless, transthoracic esophagectomy has been widely used in China, but the indication of surgical procedures regarding the left and right thoracic approaches is still controversial. In addition, the impact of surgical approaches on prognosis for the elder patients has not been discussed in detail. With the lack of an available surgical treatment strategy for esophageal cancer in the elderly patients, we aimed to examine the surgical therapy modalities and outcomes of this disease particularly for patients aged 70 years and older.

## Method

### Study Population and Data Collection

From January 2009 to October 2012, a total of 1432 consecutive patients with esophageal squamous cell carcinoma (ESCC) underwent curative resection at Sun Yat-sen University Cancer Center. The exclusion criteria were as follows: (1) patients who underwent the Ivor Lewis procedure or for whom the number of removed lymph nodes (LNs) was <15, (2) patients with a history of concurrent malignant disease or clinical T4 (tumor) staging, (3) patients who received neoadjuvant chemoradiation therapy, and (4) patients who were lost to follow up. The final study population comprised 820 patients. All patient characteristics were recorded, including demographic data, preoperative examination results, operation-related factors, cancer-specific data, and postoperative complications. Written informed consent was obtained from all patients. This study was approved by the Ethics Committee of Sun Yat-sen University Cancer Center (approval number: GZR 2018-120).

### Surgical Technique

Patients with tumor located in upper third of the esophagus were also included in our study. As for patients had upper thoracic EC, McKeown esophagectomy would be performed to ensure the resection margin free. In addition, the surgical procedure for patients with middle and lower thoracic esophageal cancer was mostly based on preoperative assessment and the preference of the surgeons. In the Sweet approach, a left posterolateral thoracotomy was performed through the fifth or sixth intercostal incision. Once the esophagus was completely dissociated, the diaphragm was incised to access and expose the abdominal cavity. An anastomosis was performed above or below the aortic arch. In the three-incision approach, a right posterolateral thoracotomy was performed initially, allowing for resection of the esophagus and mediastinal lymphadenectomy. Afterward, an abdominal incision was made for mobilization of the stomach. A left-sided cervical incision was performed for the anastomosis.^11^ Anastomoses were performed with a circular stapling device or a double layer of hand-sewn running suture. For the McKeown esophagectomy, the thoracic lymphatics were resected through the superior and posterior mediastinum, including the periesophageal, right, and left recurrent laryngeal nerve, and subcarinal nodes were completely dissected. In the abdominal nodal dissection, the upper abdominal LNs were removed, which contained splenic, common hepatic, left gastric, lesser curvature, and cardia nodes. Cervical lymphadenectomy would be carried out only if the preoperative cervical ultrasound or CT scanning presented the probability of cervical LN metastases. For the Sweet approach, LN resection in the mediastinum and abdomen was routinely performed. The pathological tumor stage and LN involvement were evaluated according to the eighth edition of the Union for International Cancer Control and the American Joint Committee on Cancer tumor node metastasis (TNM) classification.^12^

### Follow-Up of Participants

Patients were recommended for follow-up examinations at our outpatient department every 3 months for the first 2 years, every 6 months for the following 3 years, and annually thereafter. The endpoint of the study was overall survival (OS). Overall survival was defined as the number of days between the date of diagnosis and the date of any-cause death or the date of the last follow-up. Disease-free survival (DFS) was defined as the time from radical esophagectomy (R0 resection) to the first local recurrence or distant metastasis of EC. Follow-up of patients in the present study was performed until December 2018. The mean follow-up time was 43.71 months (range, 2-92 months).

### Statistical Analysis

**Figure 1.**
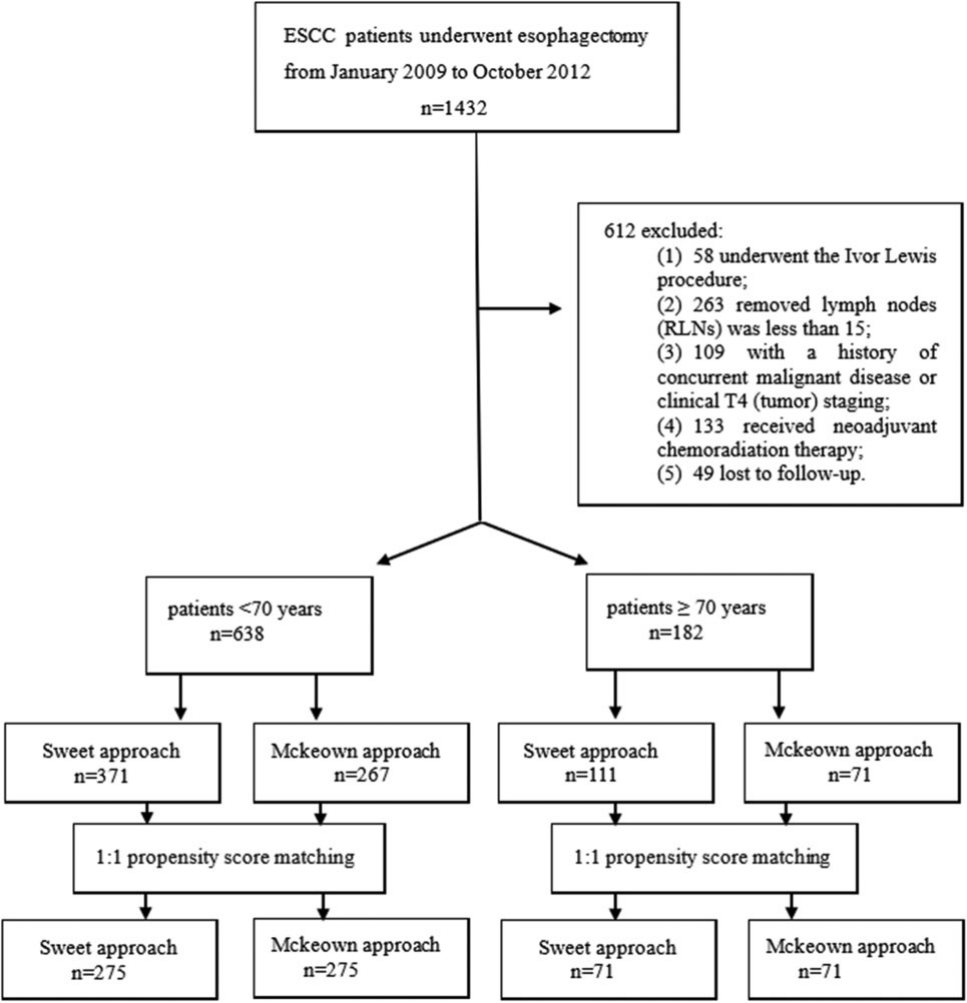
Flow diagram showing inclusion and exclusion criteria.

Propensity score matching (PSM) was used to balance the clinical characteristics of patients which received different surgical approach. Propensity scores were calculated using logistic regression and were based on gender, tumor location, tumor length, LN counts, T stage, N (LN) stage, pathological TNM stage, and adjuvant treatment. A 1:1 match was achieved using the nearest neighbor-matching algorithm with a caliper definition of 0.02.^13^ Figure 1 presents the enrollment protocol. The χ^2^ test was used to compare the categorical variables. Analysis of variance was used for the comparison of continuous variables. The survival curves were plotted using the Kaplan-Meier method. Multivariate analysis with a Cox proportional hazards model was carried out to identify significant prognostic factors. All calculations were performed using SPSS 17.0 software (SPSS, Chicago, Illinois) and R (version 3.3.0; http://www.Rproject.org), and a *P* value < .05 was considered significant.

## Results

### Patient Characteristics

After PSM, significant differences between patients younger than 70 years undergoing Sweet and McKeown approaches were still observed for tumor location, grade of differentiation, and the number of resected LNs. For patients older than 70 years before matching, significant differences were also found in the tumor location, grade of differentiation, and the LN counts between the Sweet and the McKeown procedures. After matching, only the number of removed LNs remained significantly different between the two groups. Finally, 275 and 71 paired cases were matched among patients younger and older than 70 years, respectively. Details of patient characteristics before and after matching are presented in Supplemental table 1 and Table 1.

**Table 1.** Comparison of Patient Characteristics After Propensity Score Matching Between the Sweet and the McKeown Approaches.^a^

Demographics	All Patients (*n*) (%)	Patients < 70 Years		*P*	All Patients (*n*) (%)	Patients ≥ 70 Years		*P*
		Sweet Approach	McKeown Approach			Sweet Approach	McKeown Approach	
Number	550	275	275		142	71	71	
Age (years)	57.09 ± 7.38	57.00 ± 7.51	57.19 ± 7.21	.719	74.40 ± 2.86	74.38 ± 2.73	33.49 ± 16.62	.434
Gender				.333				1.000
Female	107 (19.5)	51 (18.5)	56 (20.4)		40 (28.2)	20 (28.2)	20 (28.2)	
Male	443 (80.5)	224 (81.5)	219 (79.6)		102 (71.8)	51 (71.8)	51 (71.8)	
Location				<.001				.051
Upper third	60 (10.9)	8 (2.9)	52 (18.9)		30 (12.1)	9 (12.7)	21 (29.6)	
Middle third	232 (42.2)	108 (39.3)	124 (45.1)		63 (44.4)	35 (49.3)	28 (39.4)	
Lower third	258 (46.9)	159 (57.8)	99 (36.0)		49 (34.5)	27 (38.0)	22 (31.0)	
T stage				.402				.585
1	78 (14.1)	25 (9.1)	53 (19.3)		9 (6.3)	6 (8.5)	3 (4.2)	
2	102 (18.5)	45 (16.4)	57 (20.7)		29 (20.4)	14 (19.7)	15 (21.1)	
3	370 (67.3)	205 (74.5)	165 (60.0)		104 (73.2)	51 (71.8)	53 (74.6)	
N stage				.782				.463
0	274 (49.8)	137 (49.8)	137 (49.8)		20 (49.3)	34 (47.9)	36 (50.7)	
1	141 (25.6)	71 (25.8)	70 (25.5)		47 (33.1)	21 (29.6)	26 (36.6)	
2	100 (18.2)	47 (17.1)	53 (19.3)		19 (13.4)	12 (16.9)	7 (9.9)	
3	35 (6.4)	20 (7.3)	15 (5.5)		6 (4.2)	4 (5.6)	2 (2.8)	
Grade				<.001				.978
0	23 (4.2)	1 (0.4)	22 (8.0)		0	0	0	
1	93 (16.9)	61 (22.2)	32 (11.6)		36 (25.4)	18 (25.4)	18 (25.4)	
2	272 (49.5)	137 (49.8)	135 (49.1)		73 (51.4)	36 (50.7)	37 (52.1)	
3	162 (29.5)	76 (27.6)	86 (31.3)		33 (23.2)	17 (23.9)	16 (22.5)	
TNM staging				.632				.774
I	32 (5.7)	4 (1.4)	28 (10.1)		3 (2.1)	2 (2.8)	1 (1.4)	
II	167 (30.4)	86 (31.3)	81 (29.5)		54 (38.0)	27 (38.0)	27 (38.0)	
III	316 (57.5)	165 (60.0)	151 (54.9)		79 (55.6)	38 (35.5)	41 (57.7)	
IV	35 (6.4)	20 (7.3)	15 (5.5)		6 (4.2)	4 (5.6)	2 (2.8)	
LN resected	29.19 ± 12.39	23.37 ± 6.67	35.00 ± 13.98	<.001	29.13 ± 13.79	24.77 ± 8.28	33.49 ± 16.62	<.001
Tumor size (cm)	3.66 ± 1.58	3.71 ± 1.56	3.61 ± 13.98	.328	3.84 ± 1.56	3.87 ± 1.51	3.81 ± 1.62	.535
Adjuvant therapy				.193				.500
No	325 (59.1)	157 (57.1)	168 (61.1)		125 (88.0)	63 (88.7)	62 (87.3)	
Yes	225 (40.9)	118 (42.9)	107 (38.9)		17 (12.0)	8 (11.3)	9 (12.7)	

Abbreviation: LN, lymph node.

^a^ Data are mean ± SD or *n* (%).

### Survival

**Figure 2.**
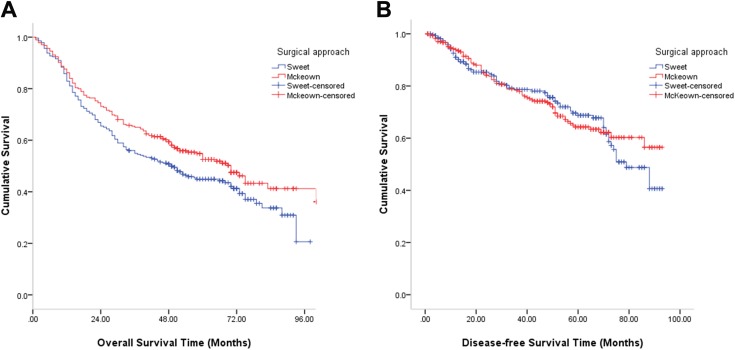
A, Overall survival in the cohort compared between the Sweet and the McKeown esophagectomy in patients younger than 70 years after propensity score matching (log rank = 4.467; *P* = .035). B, Disease-free survival in the cohort compared between the Sweet and the McKeown esophagectomy in patients younger than 70 years after propensity score matching (log rank = 0.039; *P* = .844).

**Figure 3.**
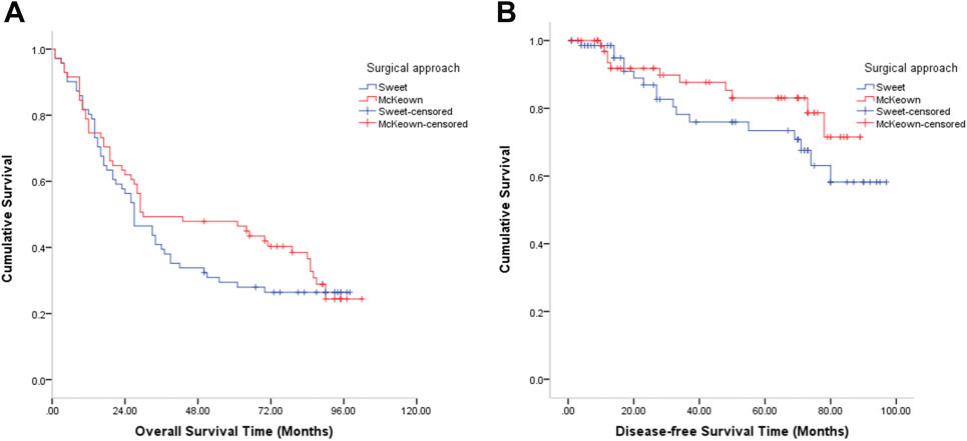
A, Overall survival in the cohort compared between the Sweet and the McKeown esophagectomy in patients older than 70 years after propensity score matching (log rank = 0.668; *P* = .414). B, Disease-free survival in the cohort compared between the Sweet and the McKeown esophagectomy in patients older than 70 years after propensity score matching (log rank = 1.562; *P* = .211).

During the follow-up period, there were 399 overall deaths in total among the 692 patients after PSM. The 5-year cumulative survival rates for patients younger than 70 years who underwent the Sweet and McKeown approaches were 44.9% and 52.1%, respectively. In addition, the 5-year cumulative survival rates for the elderly patients (≥70 years) were 28.2% and 45.5% for Sweet and McKeown groups, respectively. Kaplan-Meier analyses using log-rank test showed that patients younger than 70 years had no significant differences in DFS (log rank = 0.039; *P* = .844) between the two different surgical approaches, but the patients who underwent three-incision resection had better OS than the patients in the Sweet group (log rank = 4.467; *P* = .035) (Figure 2). However, there was no significant difference in DFS and OS between the two approaches for elderly patients (log rank = 1.562; *P* = .211 and log rank = 0.668; *P* = .414, respectively) (Figure 3).

Regression analysis using a multivariable Cox proportional hazards model revealed that tumor stage, N stage, and LN counts were independent prognostic factors in patients younger than 70 years after PSM (Table 2). Furthermore, adjuvant therapy was an independent factor for better DFS (Table 3). In particular, the McKeown approach was presented to be a positive prognostic factor compared to the Sweet in univariable analysis (HR = 0.790; 95% CI, 0.625-0.997; *P* = .047) (Supplemental table 2). However, after adjustment for other confounders, McKeown approach did not show the significant association with the prognosis (*P* > .005) (Table 2). Additionally, for patients older than 70 years, tumor length and higher N stage were found to be an independent risk prognostic factor after PSM. In contrast, lower tumor location and more resected LNs were associated with better OS (Table 2). Similarly, tumor size was related to a poor DFS (Table 3). Surgical approach was not related with prognosis significantly for the elderly patients. Univariate analysis is shown in Supplemental tables 2 and 3.

**Table 2.** Multivariate Cox Regression Analysis of Prognostic Factors Influencing Overall Survival After Propensity Score Matching.

Variables	Patients < 70 Years			Patients ≥ 70 Years		
	HR	95% CI	*P*	HR	95% CI	*P*
Gender						
Female	1			1		
Male	1.208	0.869 to 1.680	.260	1.662	0.985 to 2.805	.057
Location						
Upper third	1			1		
Middle third	0.921	0.613 to 1.383	.691	0.609	0.345 to 1.074	.087
Lower third	0.819	0.542 to 1.237	.342	0.534	0.311 to 0.916	.023
T stage						
1	1			1		
2	1.645	0.951 to 2.845	.075	1.991	0.562 to 7.054	.286
3	1.898	1.143 to 3.152	.013	2.776	0.847 to 9.093	.092
N stage						
0	1			1		
1	1.932	1.411 to 2.644	<.001	1.388	0.848 to 2.271	.192
2	3.380	2.411 to 4.739	<.001	3.967	2.143 to 7.344	<.001
3	5.534	3.608 to 8.489	<.001	8.260	2.901 to 23.519	<.001
Surgical approach						
Sweet	1			1		
McKeown	0.995	0.758 to 1.306	.973	1.053	0.663 to 1.674	.825
LN resected	0.982	0.971 to 0.994	.004	0.979	0.960 to 0.997	.026
Tumor size (cm)	1.043	0.961 to 1.132	.315	1.171	1.012 to 1.356	.034
Adjuvant therapy						
No	1			1		
Yes	0.730	0.597 to 2.349	.787	0.925	0.510 to 1.677	.798

Abbreviations: CI, confidence interval; HR, hazard ratio; LN, lymph node.

**Table 3.** Multivariate Cox Regression Analysis of Prognostic Factors Influencing Disease-Free Survival After Propensity Score Matching.

Variables	Patients < 70 Years			Patients ≥ 70 Years		
	HR	95% CI	*P*	HR	95% CI	*P*
Gender						
Female	1			1		
Male	1.196	0.765 to 1.870	.432	0.490	0.171 to 1.401	.183
Location						
Upper third	1			1		
Middle third	1.111	0.669 to 1.844	.684	0.737	0.252 to 2.149	.576
Lower third	0.882	0.529 to 1.471	.629	0.308	0.085 to 1.117	.073
N stage						
0	1			1		
1	1.194	0.797 to 1.789	.389	1.612	0.621 to 4.184	.327
2	1.351	0.870 to 2.098	.180	2.427	0.850 to 7.182	.096
3	1.840	0.998 to 3.391	.051	1.592	0.905 to 2.73	.053
Tumor size (cm)	1.071	0.972 to 1.180	.167	1.425	1.039 to 1.953	.028
Adjuvant therapy						
No	1			1		
Yes	0.570	0.370 to 0.385	<.001	0.855	0.604 to 1.726	.415

Abbreviations: CI, confidence interval; HR, hazard ratio; LN, lymph node.

### Perioperative Outcomes and Recurrence

The perioperative comparisons are presented in Table 4. For younger patients, the McKeown approach resulted in more hospitalization expenses (¥98 544.79 vs ¥67 036.77; *P* = .001), longer surgery time (463.49 minutes vs 244.87 minutes; *P* < .001), and postoperative hospital stays (22.1 days vs 9.48 days; *P* = .002) than the Sweet approach, but the blood loss was similar (227.53 mL vs 173.65 mL; *P* = .488). Additionally, more complications, especially anastomotic fistula, occurred in the McKeown group after PSM (14.5%) (Table 4). For patients older than 70 years, the three-incision procedure led to longer surgery time (460.65 minutes vs 234.21 minutes; *P* < .001), postoperative hospital stays (25.97 days vs 16.82 days; *P* = .009), and greater hospitalization expenses (¥115 283.59 vs ¥73 800.02; *P* = .034) than the Sweet approach. Additionally, the occurrence of anastomotic fistula increased in elderly patients who underwent the McKeown procedure after PSM (23.9% vs 11.3%, *P* = .038, for the McKeown and Sweet esophagectomy, respectively) (Table 4). Recurrence was observed in 188 patients of all age. After PSM, the recurrence rate between two approaches was similar in the entire cohort (patients < 70 years: 28.7% and 29.5%; patients ≥ 70 years: 23.9% and 15.4%, for the Sweet and McKeown approaches, respectively). Additionally, 19 patients died during the operative period, but no differences were found between the Sweet and the McKeown groups (deaths in patients < 70 years: 5 and 11; deaths in patients ≥ 70 years: 2 and 1, for Sweet and McKeown approaches, respectively) (Table 4).

**Table 4.** Comparison of Postoperative Consequences After Propensity Score Matching Between the Sweet and the McKeown Approaches.^a^

Variables	Patients < 70 Years			Patients ≥ 70 Years		
	Sweet	McKeown	*P*	Sweet	McKeown	*P*
Operative time (minutes)	244.87 ± 463.49	463.49 ± 129.70	<.001	234.21 ± 65.63	460.65 ± 107.73	<.001
Blood loss (mL)	173.65 ± 123.85	227.53 ± 113.72	.488	170.18 ± 117.21	230.00 ± 135.40	.547
Hospital stays (D)	9.48 ± 66.4	22.12 ± 17.98	.002	16.82 ± 13.05	25.97 ± 18.93	.009
Hospitalization expense (¥)	67 036.77	98 544.79	.001	73 800.02	11 5283.59	.034
Perioperative death, *n* (%)	5 (1.8)	11 (4.0)	.102	2 (2.8)	1 (1.4)	1.000
Recurrence, *n* (%)	79 (28.7)	81 (29.5)	.925	17 (23.9)	11 (15.3)	.292
Complications						
Anastomotic fistula	15 (5.5)	40 (14.5)	<.001	8 (11.3)	17 (23.9)	.038
Respiratory failure	5 (1.8)	11 (4.0)	.072	2 (2.8)	1 (1.4)	1.000
Pneumonia	7 (2.5)	19 (6.9)	.007	1 (1.4)	2 (2.8)	.597
Chylothorax	1 (0.4)	8 (2.9)	.013	0	0	

^a^ Data are mean, mean ± SD, or *n* (%).

## Discussion

Esophagectomy is considered to be the most effective treatment for patients with ESCC where surgery is possible, while it also contributes to a relatively high incidence of complications. Therefore, the most appropriate surgical approach for esophagectomy is still uncertain, especially for elderly patients. A previous study reported that there were nearly 33.1% of elderly patients who did not receive treatment after diagnosis, despite the fact that increased operative adverse events and mortality in elderly patients with greater comorbidities may result in the poor survival outcomes.^14^ Oncology does not have a specific age threshold for elderly patients with cancer. As in previous reports of ESCC, elderly patients’ age was defined as ≥70 years.^14-16^ Therefore, the present study stated a cutoff age threshold of 70 years to define the elderly patients’ cohort. The McKeown and Sweet procedures have been widely performed to remove the tumor in our center since 2009. This study compared the perioperative outcomes and long-term survival rates of two surgical approaches in patients with esophageal cancer younger than 70 years or older than 70 years.

Regarding to the comparisons between different surgical approaches for patients with EC, several studies have investigated the short- and long-term outcomes of patients who underwent either the Ivor-Lewis or Sweet procedures.^17-20^ However, most of the randomized clinical trials did not discuss the comparison between the McKeown and the Sweet esophagectomy, and patients older than 75 years were usually not included for analysis,^18,20^ which resulted in the lack of an indication about the appropriate procedure for the elderly patients with ESCC.

The two most common surgical approaches in our cancer center are the Sweet and McKeown procedures. The Sweet approach was first described by Churchill and Sweet in 1942.^21^ It offers adequate exposure of the hiatus and stomach with a single incision, which benefits patients with tumors in the middle and lower third of the esophagus. The three-incision approach was proposed by McKeown in 1976.^11^ It is more convenient for extended lymphadenectomy and benefits for patients with positive LNs, especially for the LNs located in the upper mediastinal region. The McKeown esophagectomy is advocated by the Chinese surgeons for its radical dissection of the left and right recurrent laryngeal nerve nodes, which ensures accurate pathological staging.^22^ However, the Sweet approach with limited lymphadenectomy still predominates with the three-incision procedure being associated with higher postoperative complications. According to the NCCN guidelines for the treatment of esophageal and esophagogastric junction cancers, at least 15 nodes should be removed in radical resection for esophageal cancer.^22^ Complete resection of the esophagus and regional LNs is essential to improve long-term survival.^23^ To avoid inaccurate LN dissection, which may result in inappropriate pathologic nodal staging and treatment, a phenomenon called stage migration,^24^ our study only included patients who had more than 15 LNs removed. The present study suggests that more LNs count was independently associated with higher OS for all patients (Table 3). The McKeown approach could resect more LNs than the Sweet approach after PSM (mean ± SD: patients < 70 years: 23.37 ± 6.67 vs 35.00 ± 13.98, *P* < .001 and patients ≥ 70 years: 24.77 ± 8.28 vs 33.49 ± 16.62, *P* < .001, for the Sweet and McKeown approaches, respectively). More importantly, our study found that for patients younger than 70 years, McKeown esophagectomy could contribute to a better OS than the Sweet approach (median survival time: 70 months vs 49 months), even though the three-incision procedure resulted in a longer operative time and a higher incident rate of complications. However, for the elderly patients, the McKeown approach with extended lymphadenectomy did not seem to be beneficial for a better OS when the adequate number of LNs was resected with Sweet procedure (median survival time: 30 months vs 27 months, for McKeown and Sweet esophagectomy, respectively).

Previous studies have demonstrated that the 5-year survival rate of elderly patients ranged from 21% to 47%.^16,25^ The present study found that after surgical resection, the overall 5-year survival rate of patients with ESCC older than 70 years was 37.3%. Patients older than 70 years had the higher incidence rate of postoperative complications and operative and in-hospital mortality.^16,26^ Additional abdominal and neck incisions are required for the McKeown when compared to the Sweet approach, which could lead to the increased operative times, blood loss, wound infection rates, and length of hospitalization. In our study, for elderly patients, the blood loss during the operation was not significantly different between left and right transthoracic esophagectomy, whereas the latter resulted in the longer operation times and hospital stays (Table 4). In addition, the occurrence of anastomotic fistula did increase in patients older than 70 years who underwent the McKeown procedure (23.9% vs 11.3%, for the McKeown and Sweet esophagectomy, respectively) (Table 4). Anastomotic leakage is a severe complication that can be fatal. Therefore, we recommended that the younger patients with EC who are in good cardiopulmonary condition should undergo the McKeown esophagectomy for better LN resection, which could provide more accurate pathological staging and lead to a favorable prognosis. Additionally, the Sweet approach should be considered for patients older than 70 years. Our study demonstrated that elderly patients in the Sweet group experienced similar outcomes compared with those in the McKeown group when more than 15 LNs were guaranteed to be removed. We hypothesize that the single-incision approach contributes to reduce surgical trauma and the rate of anastomotic leakage, which benefits the prognosis of elderly patients.

It should be noted that patients who received neoadjuvant therapy were excluded from the analysis. Yang et al^27^ reported that neoadjuvant CRT followed by surgery could improve survival among patients with locally advanced ESCC, however, none of the patients were older than 70 years in the randomized clinical trial. The optimal neoadjuvant treatment regimen has not been established, and the role of neoadjuvant therapy for the elderly patients is unclear. Additionally, there is no general consensus about postoperative treatment for the elderly patients, and only 17 (12.0%) patients received adjuvant therapy after the operation in the current study. The treatment regimen was determined by the doctor subjectively, to some extent, considering the pathological staging and performance status of each patients comprehensively. We found some elderly patients who were ineligible to receive adjuvant treatment because of poor physical recovery after the aggressive operation, even in advanced disease, which may be the reason for the low rate of postoperative therapy in our study.

To provide more information on outcomes of esophagectomy in the elderly patients with ESCC, we used data from a single center. To our knowledge, this is the first study to describe the younger and older patients with ESCC who have undergone either the Sweet or the McKeown procedure, respectively, and analyzing these patients after a 1:1 PSM to minimize selection bias. Our study had some limitations. First, its retrospective design may result in some statistical biases, and the option of surgical approaches was determined based on the experience of the surgeon, and the patients were not randomized. Second, we did not analyze the incidence of recurrent nerve palsy because it rarely happened to a patient who underwent the Sweet approach. In addition, the effect of the Ivor Lewis, minimally invasive approach, and adjuvant therapy in elderly patients with ESCC is still unclear, due to the limited sample size of the elder patients who received the treatments. Another limitation of the current study was that we did not evaluate the postoperative quality of life, which might be associated with patient outcomes.

## Conclusion

Our study provides evidence for the superiority of the McKeown approach with regard to extended lymphadenectomy and accurate staging which increases the OS in younger patients with ESCC. However, for patients older than 70 years, the Sweet approach was proven to be an effective therapy considering the better perioperative outcomes and similar long-term survival compared with patients in the McKeown group. Further randomized clinical trials are needed in the future to conclude the optimal treatment protocol for the elderly patients with ESCC.

## Supplemental Material

supplemental_table_1 - Comparison of Outcomes Between McKeown and Sweet Esophagectomy in the Elderly Patients for Esophageal Squamous Cell Carcinoma: A Propensity Score-Matched Analysis Click here for additional data file. supplemental_table_1 for Comparison of Outcomes Between McKeown and Sweet Esophagectomy in the Elderly Patients for Esophageal Squamous Cell Carcinoma: A Propensity Score-Matched Analysis by Dongni Chen, Yihuai Hu, Youfang Chen, Jia Hu and Zhesheng Wen in Cancer Control

supplmental_table_2 - Comparison of Outcomes Between McKeown and Sweet Esophagectomy in the Elderly Patients for Esophageal Squamous Cell Carcinoma: A Propensity Score-Matched Analysis Click here for additional data file. supplmental_table_2 for Comparison of Outcomes Between McKeown and Sweet Esophagectomy in the Elderly Patients for Esophageal Squamous Cell Carcinoma: A Propensity Score-Matched Analysis by Dongni Chen, Yihuai Hu, Youfang Chen, Jia Hu and Zhesheng Wen in Cancer Control

supplmental_table_3 - Comparison of Outcomes Between McKeown and Sweet Esophagectomy in the Elderly Patients for Esophageal Squamous Cell Carcinoma: A Propensity Score-Matched Analysis Click here for additional data file. supplmental_table_3 for Comparison of Outcomes Between McKeown and Sweet Esophagectomy in the Elderly Patients for Esophageal Squamous Cell Carcinoma: A Propensity Score-Matched Analysis by Dongni Chen, Yihuai Hu, Youfang Chen, Jia Hu and Zhesheng Wen in Cancer Control
