# Supplementary material for: Comparison of Outcomes Between McKeown and Sweet Esophagectomy in the Elderly Patients for Esophageal Squamous Cell Carcinoma: A Propensity Score-Matched Analysis
Source: Cancer Control. 2020 Feb 12;27(1):1073274820904700. doi: 10.1177/1073274820904700 (PMC7020469; doi:10.1177/1073274820904700)
Supplement: supplemental_table_1 - Comparison of Outcomes Between McKeown and Sweet Esophagectomy in the Elderly Patients for Esophageal Squamous Cell Carcinoma: A Propensity Score-Matched Analysis [file supplemental_table_1.pdf]

Supplemental table 1: Comparison of patient characteristics before propensity score matching between the Sweet and McKeown approaches

Demographics	Patients< 70 years			*P*	Patients ≥70 years			*P*
	All patients (n) (%)	Sweet approach	McKeown approach		All patients (n) (%)	Sweet approach	McKeown approach	
**Number**	638	371	267		182	111	71	
**Age (y)**	56.65±7.29	56.39±7.49	56.66±7.02	0.330	57.09±7.38	57.00±7.51	57.19±7.21	0.719
**Gender**				0.286				0.207
Female	125(19.0)	76(20.5)	49(18.4)		59(32.4)	39(35.1)	20(28.2)	
Male	513(80.4)	295(79.5)	218(81.6)		123(67.6)	72(64.9)	51(71.8)	
**Location**				<0.001				0.001
Upper third	69(10.8)	12(3.2)	57(21.3)		31(17.0)	10(9.0)	21(29.6)	
Middle third	258(40.4)	144(38.8)	114(42.7)		73(40.1)	45(40.5)	28(39.4)	
Lower third	311(48.7)	215(58.0)	96(36.0)		78(42.9)	56(50.5)	22(31.0)	
**T stage**				0.457				0.798
1	76(11.9)	41(11.1)	35(13.1)		10(5.5)	7(6.3)	3(4.2)	
2	136(21.3)	75(20.2)	61(22.8)		40(22.0)	25(22.5)	15(21.1)	
3	436(66.8)	255(68.7)	171(64.0)		132(72.5)	79(71.2)	53(74.6)	
**N stage**				0.316				0.795
0	332(52.0)	203(54.7)	129(48.3)		91(50.0)	55(49.5)	36(50.7)	
1	164(25.7)	91(24.5)	73(27.3)		62(34.1)	36(32.4)	26(36.6)	
2	106(6.6)	55(14.8)	51(19.1)		23(12.6)	16(14.4)	7(9.9)	
3	36(5.6)	22(5.9)	14(5.2)		6(3.3)	4(3.6)	2(2.8)	
**Grade**				0.006				0.044
0	6(0.9)	1(0.3)	5(1.9)		0	0	0	
1	110(17.2)	77(20.8)	33(12.4)		44(24.2)	26(23.4)	18(25.4)	
2	327(51.3)	189(50.9)	138(51.7)		78(42.9)	41(36.9)	37(52.1)	
3	195(30.6)	104(28.0)	91(34.1)		60(33.0)	44(39.6)	16(22.5)	
**TNM staging**				0.727				0.855
I	15(2.4)	7(1.9)	8(3.0)		4(2.2)	3(2.7)	1(1.4)	
II	205(32.1)	116(31.3)	89(33.3)		73(40.1)	46(41.4)	27(38.0)	
III	382(59.9)	226(60.9)	156(58.4)		99(54.4)	58(52.3)	41(57.7)	
IV	36(5.6)	22(5.9)	14(5.2)		6(3.3)	4(3.6)	2(2.8)	
**LN resected**	25.37 ± 13.51	19.39±8.43	34.37 ± 14.94	<0.001	27.54 ± 12.91	23.73±7.81	33.49 ± 16.62	<0.001
**Tumor size(cm)**	3.88±5.42	3.88±5.42	3.69±1.60	0.416	3.89±1.53	3.94±1.47	3.81±1.62	0.710
**Adjuvant therapy**				0.304				0.291
No	376(58.9)	215(58.0)	161(60.3)		163(89.6)	101(91.0)	62(87.3)	
Yes	262(41.1)	156(42.0)	106(39.7)		19(10.4)	10(9.0)	9(12.7)	

Data are mean± SD or n (%).

LN= lymph node.
