# Supplementary material for: Comparison of Outcomes Between McKeown and Sweet Esophagectomy in the Elderly Patients for Esophageal Squamous Cell Carcinoma: A Propensity Score-Matched Analysis
Source: Cancer Control. 2020 Feb 12;27(1):1073274820904700. doi: 10.1177/1073274820904700 (PMC7020469; doi:10.1177/1073274820904700)
Supplement: supplmental_table_2 - Comparison of Outcomes Between McKeown and Sweet Esophagectomy in the Elderly Patients for Esophageal Squamous Cell Carcinoma: A Propensity Score-Matched Analysis [file supplmental_table_2.pdf]

Supplemental table 2. Univariate cox regression analysis of prognostic factors influencing overall survival.

	Patients<70 years			Patients≥70 years		
**Variables**	HR	95% CI	*P*	HR	95% CI	*P*
**Gender**						
Female	1			1		
Male	1.488	1.084-2.044	0.014	2.022	1.249-3.271	0.004
**Location**						
Upper third	1			1		
Middle third	1.088	0.737-1.606	0.671	0.592	0.364-0.965	0.035
Lower third	1.051	0.715-1.545	0.801	0.640	0.385-1.062	0.084
**T stage**						
1	1			1		
2	2.001	1.172-3.416	0.011	2.963	0.883-9.940	0.079
3	2.571	1.589-4.161	<0.001	3.081	0.972-9.769	0.056
**N stage**						
0	1			1		
1	2.062	1.526-2.788	<0.001	1.759	1.122-2.757	0.014
2	3.652	2.698-4.944	<0.001	5.293	2.969-9.434	<0.001
3	5.771	3.875-8.595	<0.001	12.364	4.898-31.213	<0.001
**Grade**						
0	1					
1	1.081	0.262-4.468	0.914	1		
2	1.418	0.351-5.725	0.624	0.752	0.469-1.206	0.237
3	1.587	0.391-6.440	0.518	1.431	0.850-2.410	0.178
**TNM staging**						
I	1			1		
II	1.905	0.698-5.194	0.208	1.403	0.338-5.827	0.641
III	1.968	0.730-5.310	0.181	1.595	0.389-6.536	0.517
IV	6.520	2.310-18.405	<0.001	10.650	2.076-54.626	0.005
**LN resected**	0.988	0.978-0.998	0.017	0.975	0.959-0.992	0.004
**Tumor size(cm)**	1.121	1.045-1.201	0.001	1.058	0.932-1.201	0.382
**Surgical approach**						
Sweet	1			1		
McKeown	0.790	0.625-0.997	0.047	0.852	0.577-1.257	0.419
**Adjuvant therapy**						
No	1			1		
Yes	0.704	0.490-0.876	<0.001	1.116	0.690-2.142	0.499

HR=hazard ratio; CI=confidence interval; LN= lymph node.
