# Supplementary material for: Comparison of Outcomes Between McKeown and Sweet Esophagectomy in the Elderly Patients for Esophageal Squamous Cell Carcinoma: A Propensity Score-Matched Analysis
Source: Cancer Control. 2020 Feb 12;27(1):1073274820904700. doi: 10.1177/1073274820904700 (PMC7020469; doi:10.1177/1073274820904700)
Supplement: supplmental_table_3 - Comparison of Outcomes Between McKeown and Sweet Esophagectomy in the Elderly Patients for Esophageal Squamous Cell Carcinoma: A Propensity Score-Matched Analysis [file supplmental_table_3.pdf]

Supplemental table 3. Univariate cox regression analysis of prognostic factors influencing disease-free survival.

	Patients<70 years			Patients≥70 years		
**Variables**	HR	95% CI	*P*	HR	95% CI	*P*
**Gender**						
Female	1			1		
Male	1.607	1.032-2.506	0.036	1.342	0.543-3.318	0.524
**Location**						
Upper third	1			1		
Middle third	1.111	0.669-1.844	0.684	1.281	0.500-3.279	0.606
Lower third	0.882	0.529-1.471	0.629	0.583	0.188-1.810	0.351
**T stage**						
1	1			1		
2	0.560	0.199-1.575	0.272	0.324	0.109-1.860	0.336
3	0.837	0.322-2.177	0.715	2.464	0.834-3.892	0.538
**N stage**						
0	1			1		
1	2.108	1.430-3.109	<0.001	1.217	0.504-2.940	0.662
2	3.353	2.228-5.047	<0.001	2.079	0.795-5.436	0.136
3	4.460	2.472-8.046	<0.001	3.119	2.173-7.146	<0.001
**Grade**						
0	1					
1	0.788	0.295-2.105	0.634	1		
2	1.310	0.530-3.237	0.558	0.548	0.227-1.325	0.182
3	1.504	0.601-3.764	0.383	0.981	0.378-2.544	0.968
**TNM staging**						
I	1			1		
II	2.547	0.620-10.464	0.195	3.409	0.710-15.921	0.310
III	2.206	0.543-8.969	0.269	4.212	0.856-9.815	0.290
IV	6.345	1.438-27.997	0.015	5.924	0.601-9.340	0.431
**LN resected**	0.989	0.976-1.002	0.104	0.986	0.959-1.014	0.336
**Tumor size(cm)**	1.118	1.020-1.225	0.017	1.136	0.898-1.436	0.287
**Surgical approach**						
Sweet	1			1		
McKeown	0.992	0.726-1.356	0.962	0.608	0.285-1.299	0.199
**Adjuvant therapy**						
No	1			1		
Yes	0.557	0.370-0.838	<0.001	0.875	0.490-1.981	0.319

HR=hazard ratio; CI=confidence interval; LN= lymph node.
